# Mitoketoscins: Novel mitochondrial inhibitors for targeting ketone metabolism in cancer stem cells (CSCs)

**DOI:** 10.18632/oncotarget.21259

**Published:** 2017-09-24

**Authors:** Bela Ozsvari, Federica Sotgia, Katie Simmons, Rachel Trowbridge, Richard Foster, Michael P. Lisanti

**Affiliations:** ^1^ Translational Medicine, School of Environment & Life Sciences, University of Salford, Greater Manchester, UK; ^2^ The Paterson Institute, University of Manchester, Withington, UK; ^3^ School of Molecular & Cellular Biology & Astbury Centre for Structural Molecular Biology, Faculty of Biological Sciences, University of Leeds, West Yorkshire, UK; ^4^ School of Chemistry, Faculty of Mathematics and Physical Sciences, University of Leeds, West Yorkshire, UK

**Keywords:** ketone bodies, drug design, mitochondria, tumor-initiating cells, cancer stem-like cells

## Abstract

Previous studies have now well-established that epithelial cancer cells can utilize ketone bodies (3-hydroxybutyrate and aceto-acetate) as mitochondrial fuels, to actively promote tumor growth and metastatic dissemination. The two critical metabolic enzymes implicated in this process are OXCT1 and ACAT1, which are both mitochondrial proteins. Importantly, over-expression of OXCT1 or ACAT1 in human breast cancer cells is sufficient to genetically drive tumorigenesis and/or lung metastasis, validating that they indeed behave as metabolic “tumor promoters”. Here, we decided to target these two enzymes, which give cancer cells the ability to recycle ketone bodies into Acetyl-CoA and, therefore, to produce increased ATP. Briefly, we used computational chemistry (*in silico* drug design) to select a sub-set of potentially promising compounds that spatially fit within the active site of these enzymes, based on their known 3D crystal structures. These libraries of compounds were then phenotypically screened for their effects on total cellular ATP levels. Positive hits were further validated by metabolic flux analysis. Our results indicated that four of these compounds effectively inhibited mitochondrial oxygen consumption. Two of these compounds also induced a reactive glycolytic phenotype in cancer cells. Most importantly, using the mammosphere assay, we showed that these compounds can be used to functionally inhibit cancer stem cell (CSC) activity and propagation. Finally, our molecular modeling studies directly show how these novel compounds are predicted to bind to the active catalytic sites of OXCT1 and ACAT1, within their Coenzyme A binding site. As such, we speculate that these mitochondrial inhibitors are partially mimicking the structure of Coenzyme A. Thus, we conclude that OXCT1 and ACAT1 are important new therapeutic targets for further drug development and optimization. We propose that this new class of drugs should be termed “*mitoketoscins*”, to reflect that they were designed to target ketone re-utilization and mitochondrial function.

## INTRODUCTION

Ketones (3-hydroxybutyrate, acetoacetate and acetone) are high-energy mitochondrial fuels; they are naturally generated by hepatocytes, during periods of caloric restriction, fasting and/or starvation [1-3]. During nutrient deprivation, ketone bodies secreted into the blood are then directed towards the brain, where neurons convert them back into Acetyl-CoA, so they can be effectively re-utilized as an energy source [1-3]. The two most critical neuronal enzymes for this ketone re-utilization process are OXCT1 and ACAT1 [2, 3], as they are directly involved in the conversion of ketone bodies into Acetyl-CoA, so that they can re-enter the TCA cycle and generate mitochondrially-derived ATP.

Recently, we showed that a similar “ketone-shuttle” also exists in human tumors, whereby ketogenic cancer-associated fibroblasts (CAFs) locally produce ketone bodies, for their re-utilization by mitochondria in adjacent human breast cancer cells [2-8]. In further support of this “metabolic-coupling” hypothesis, recombinant over-expression of ACAT1 or OXCT1 in MDA-MB-231 breast cancer cells was indeed sufficient to promote tumor growth and lung metastasis [4]. These data provide genetic evidence that ketone body re-utilization can help drive tumor progression and metastasis [4, 5].

Using a metabolo-genomics approach, we also tested the effects of ketone bodies on the transcriptional profile of MCF7 breast cancer cells [8]. Interestingly, we observed that these ketone-induced mRNA profiles were most tightly associated with (1) stemness (neural, embryonic and hematopoietic), (2) a reduction in DNA damage and (3) breast cancer (ER-negative vs. ER-positive) [8]. Most importantly, the ketone-induced “gene signature” was able to predict poor clinical outcome (including recurrence and metastasis), in ER(+) human breast cancer patients. In accordance with these findings, treatment of embryonic stem (ES) cells with ketone bodies was sufficient to enhance their “stemness phenotype”. For example, ES cell colony size (diameter) was increased up to 25%, while colony number was increased by ∼2-3-fold [8].

Based on these and other ketone-related studies, it appears that the enzymes driving ketone re-utilization, namely ACAT1 and OXCT1, would be excellent therapeutic targets for drug development, especially for the eradication of cancer stem cells (CSCs). Here, we tested this hypothesis directly, by identifying new small molecules to exploit ketone metabolism. Importantly, our results directly show that these new chemical entities, targeting ACAT1 and OXCT1, are indeed sufficient to block CSC propagation and to effectively inhibit oxidative mitochondrial metabolism.

## RESULTS

### Overall strategy for the discovery of new inhibitors of ketone re-utilization and mitochondrial metabolism

In order to identify novel metabolic inhibitors to target the re-utilization of ketone bodies as mitochondrial fuels, we took advantage of the known 3D crystal structures of the enzymes responsible for this process, namely ACAT1 and OXCT1. Both of these enzymes are mitochondrial proteins, which are involved in the chemical conversion of ketone bodies into Acetyl-CoA, which can then enter the TCA cycle. So, effective ACAT1 and OXCT1 inhibitors would be expected to phenotypically drive ATP depletion in cancer cells.

**Figure 1:**
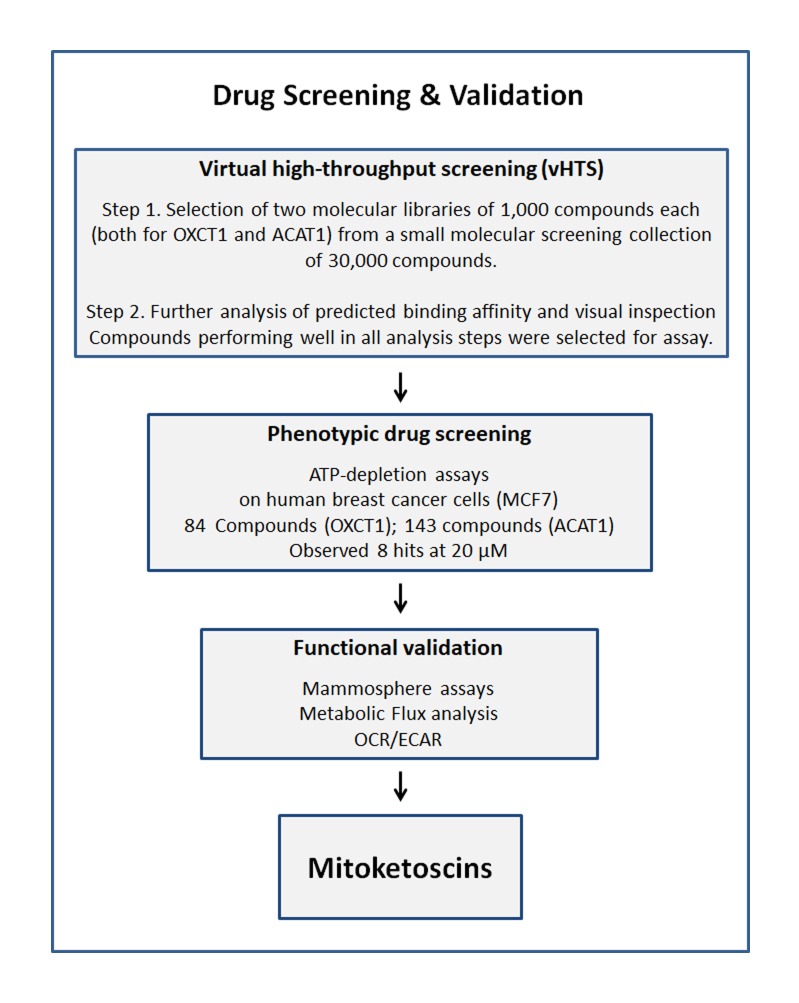
Schematic diagram illustrating our overall drug discovery strategy, employing both in silico and phenotypic drug screening 1. Virtual high-throughput screening (vHTS) - We used the 3D structure of porcine OXCT1 and human ACAT1 proteins to screen a virtual collection of 30,000 compounds and identified a subset of 1,000 compounds each that “bind” in silico. Further analysis of predicted binding affinity and visual inspection was performed. Compounds performing well in all analysis steps were then selected for assay. 2. Phenotypic drug screening - The resulting compound libraries were then subjected to phenotypic drug screening at a concentration of 20 µM, to identify which compounds functionally induce ATP-depletion, before inducing cell death. Subsequently, positive hits were re-screened at the same and lower concentrations (20 µM and 10 µM), to identify the top eight compounds that most potently induced ATP-depletion. 3. Functional validation - The top hits were then further validated using mammosphere assays (for assessing potential anti-cancer stem cell activity). Metabolic flux analysis, to determine specific effects on oxygen consumption, to estimate their anti-mitochondrial activity, and viability assays were also carried out.

Our overall screening strategy is briefly illustrated schematically in Figure 1. First, we used the 3D structure of porcine OXCT1 and human ACAT1 proteins to virtually screen an in-house library of ∼30,000 compounds and identified a subset of ∼1,000 compounds each that are predicted to bind to these proteins *in silico.* Further analysis of predicted binding affinity and visual inspection was performed. Compounds performing well in all analysis steps (an overall library of 227 compounds) were then selected for assay.

Second, the resulting compound libraries were then subjected to phenotypic drug screening at a concentration of 20 µM, to identify which compounds functionally induce ATP-depletion, before inducing cell death. Subsequently, positive hits were re-screened at the same and lower concentrations (20 µM and 10 µM), to identify the top eight compounds that most potently induced ATP-depletion.

Finally, the eight top hits were then further validated using 3D mammosphere assays to assess their potential anti-cancer stem cell activity. Metabolic flux analysis was also performed to determine their specific effects on i) mitochondrial oxygen consumption and ii) glycolytic activity.

Thus, the overall hit rate was 8 out of 30,000 (1/3,750 = 0.03%), if we include both vHTS and phenotypic screening. After further validation studies focused on anti-CSC activity, only 5 final compounds remained (with IC-50’s between 10 and 70 μM), yielding an overall hit rate of 5 out of 30,000 (1/6,000 = 0.017%). As such, this screening and validation procedure specifically excluded 99.98% of the compounds that we assessed.

### Functional and metabolic characterization of the positive hit compounds

**Figure 2:**
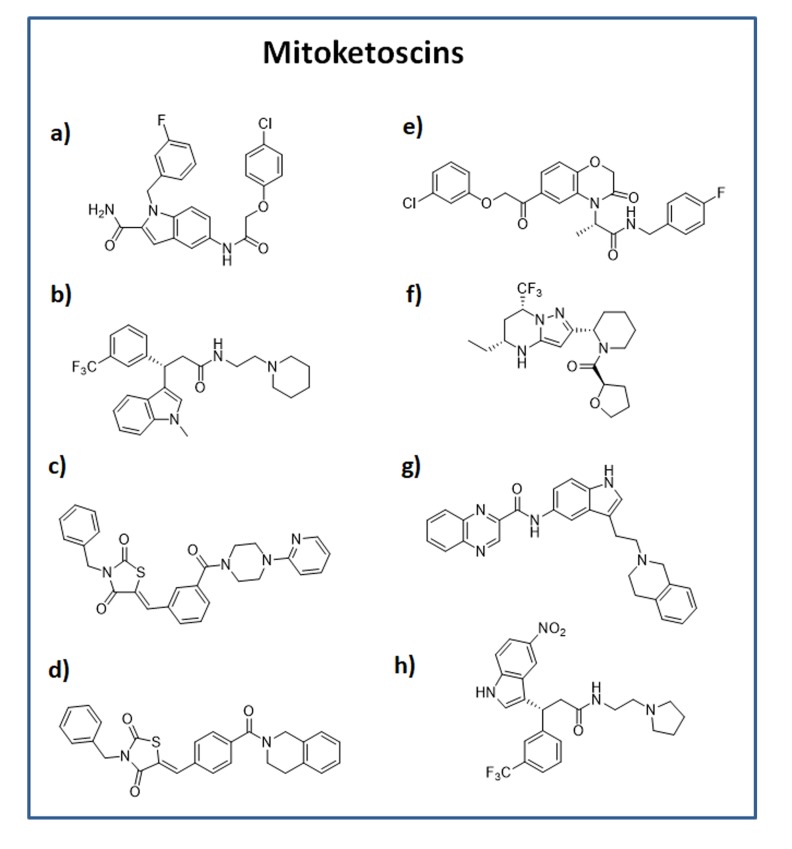
Chemical structures of the top 8 hits OXCT1 hits: **a**) compound 1 (ALB-H01004577); **b**) compound 2 (ALB-H09465625); **c**) compound 3 (ALB-H15358970); **d**) compound 4 (ALB-H15354504); ACAT1 hits: **e**) compound 5 (ALB-H04367562); **f**) compound 6 (LEG19576081); **g**) compound 7 (ALB-H10747299); **h**) compound 8 (ALB-H01005022).

The structures of the eight positive hit compounds are shown in Figure 2. More specifically, compounds 1-4 are derived from the OXCT1 screen, while compounds 5-8 are from the ACAT1 screen. Interestingly, note that compounds 2 and 8 are very structurally similar, indicating that some of these compounds could be possibly used to target both OXCT1 and ACAT1, at the same time.

**Figure 3:**
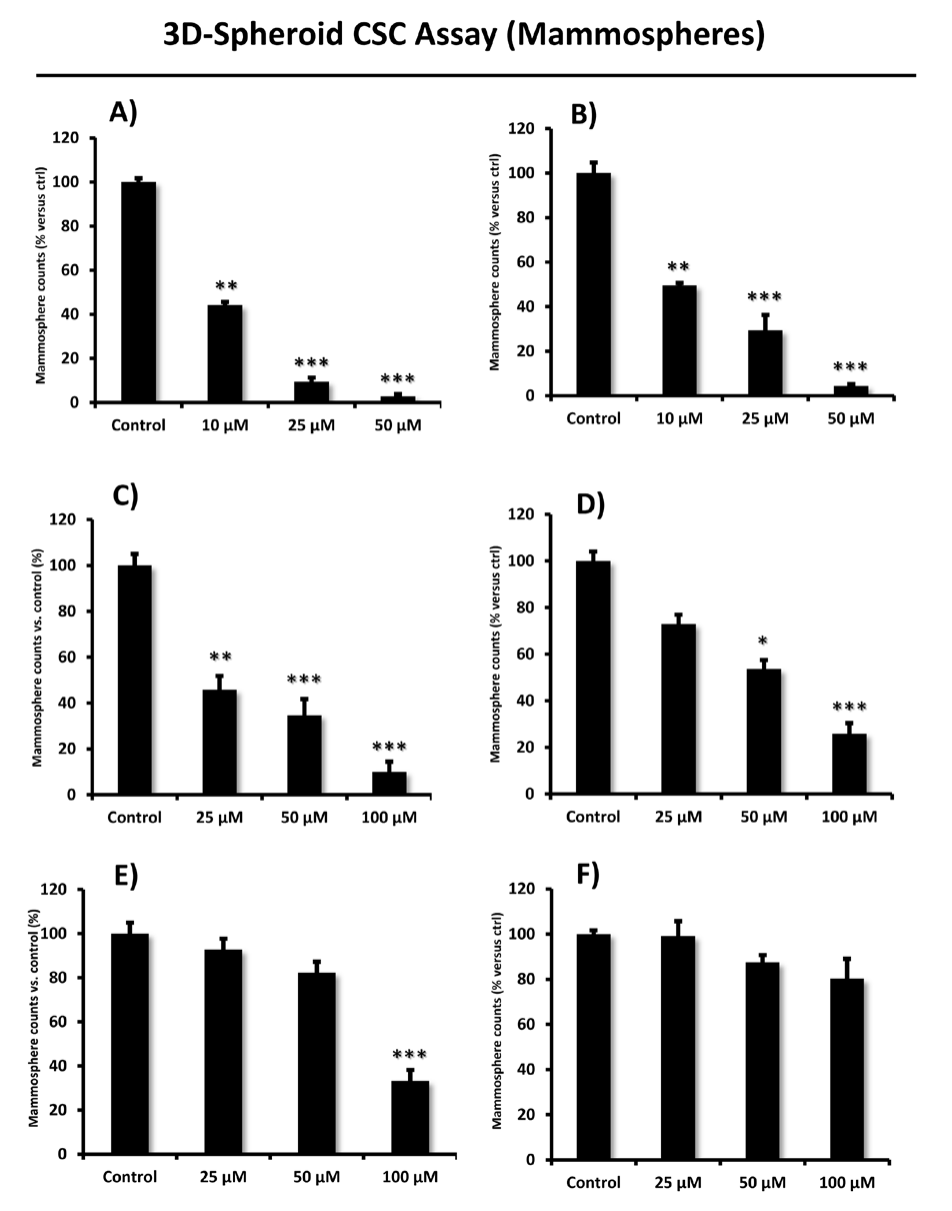
Effects of the top 6 hit compounds on mammosphere formation Compound 2 and 8 (Panel **A.** and **B.**) were the two most potent hits in decreasing the number of mammospheres, a measure of cancer stem cell activity, at a concentration of 25 µM. Compound 6 and 3 were also effective (see panel **C.** and **D.**), while compound 5 and 1 (panel **E.** and **F.**) were less potent inhibitors of mammosphere growth. Compound 4 had no effect; compound 7 was excluded in this experiment due to solubility problems. Bar graphs are shown as the mean ± SEM; t-test, two-tailed test *p < 0.05, **p < 0.005, ***p < 0.0001.

All eight compounds were next screened to functionally assess their ability to inhibit mammosphere formation, which directly measures cancer “stem cell activity”. This assay measures the ability of CSCs to undergo clonal anchorage-independent growth, under non-adhesive conditions, which is a characteristic of cells associated with metastatic potential. Six of the compounds successfully inhibited mammosphere formation, with IC-50’s between 10 and 170 μM (Figure 3). These results are also summarized in Table 1.

**Table 1:** IC-50 values for the mitoketoscins

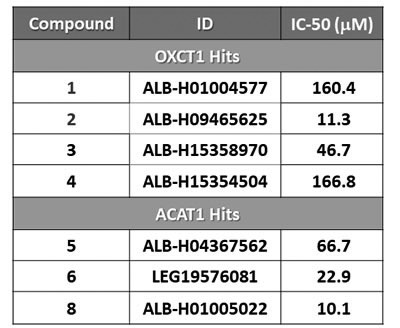

IC-50 values of top hit compounds are shown highlighting their efficacy in the 3D-spheroid assay for detecting CSC activity.

Compounds 1 to 4 are hits from the OXCT1 screen.

Compounds 5 to 8 are hits from the ACAT1 screen.

Their rank order potency was as follows: 8 > 2 > 6 > 3 > 5 > 1 > 4. Thus, we decided to focus our efforts on the metabolic characterization of the top four compounds (8 > 2 > 6 > 3), with IC-50’s between 10 and 50 μM. In this context, it is important to note that compounds 8 and 2 are nearly structurally identical, and as such functionally show extremely similar IC-50’s between 10 -to- 11 μM for targeting the propagation of CSCs.

**Figure 4:**
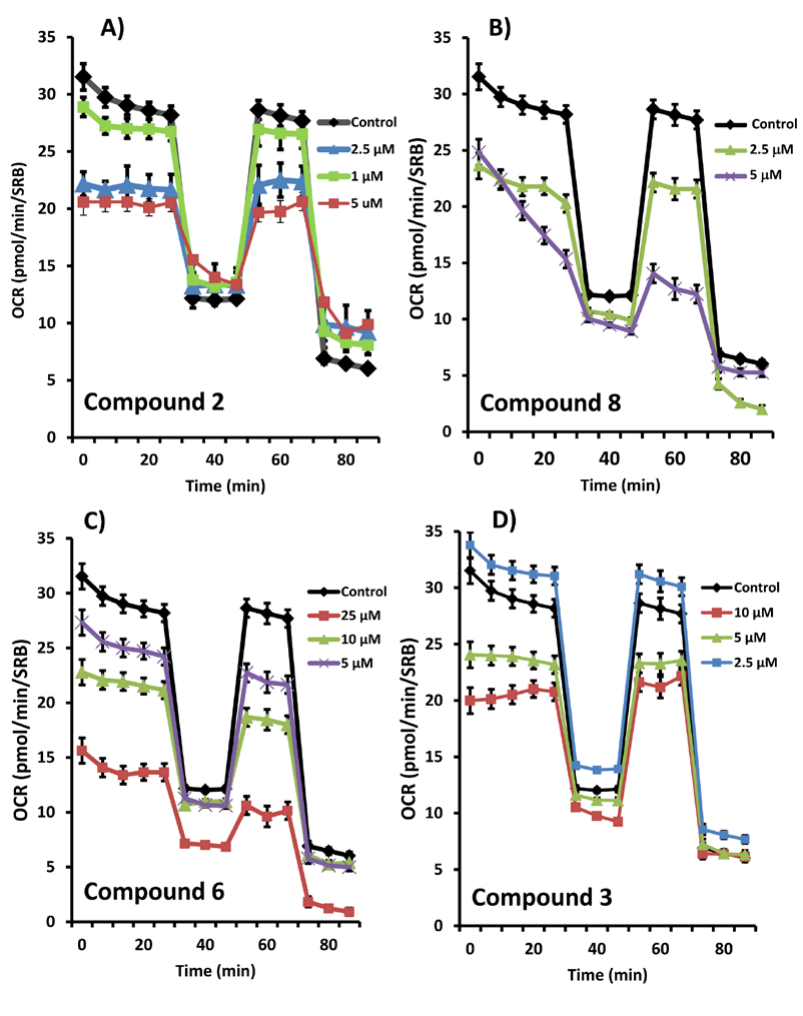
Effects of the top 4 hit compounds on the metabolic activity of MCF7 human breast cancer cells Oxygen consumption rate (OCR) was measured using the Seahorse XFe96 Metabolic Flux Analyzer. Then data were normalized to protein content (SRB assay). Note that compound 2 or 8 treatments reduced mitochondrial respiration significantly even at a dose as low as 5 µM (see panel **A.** and **B.**). Compound 6 and 3 were also potent inhibitors (panel **C.** and **D.**). MCF7 cells were treated with each compound for 72 hours.

**Figure 5:**
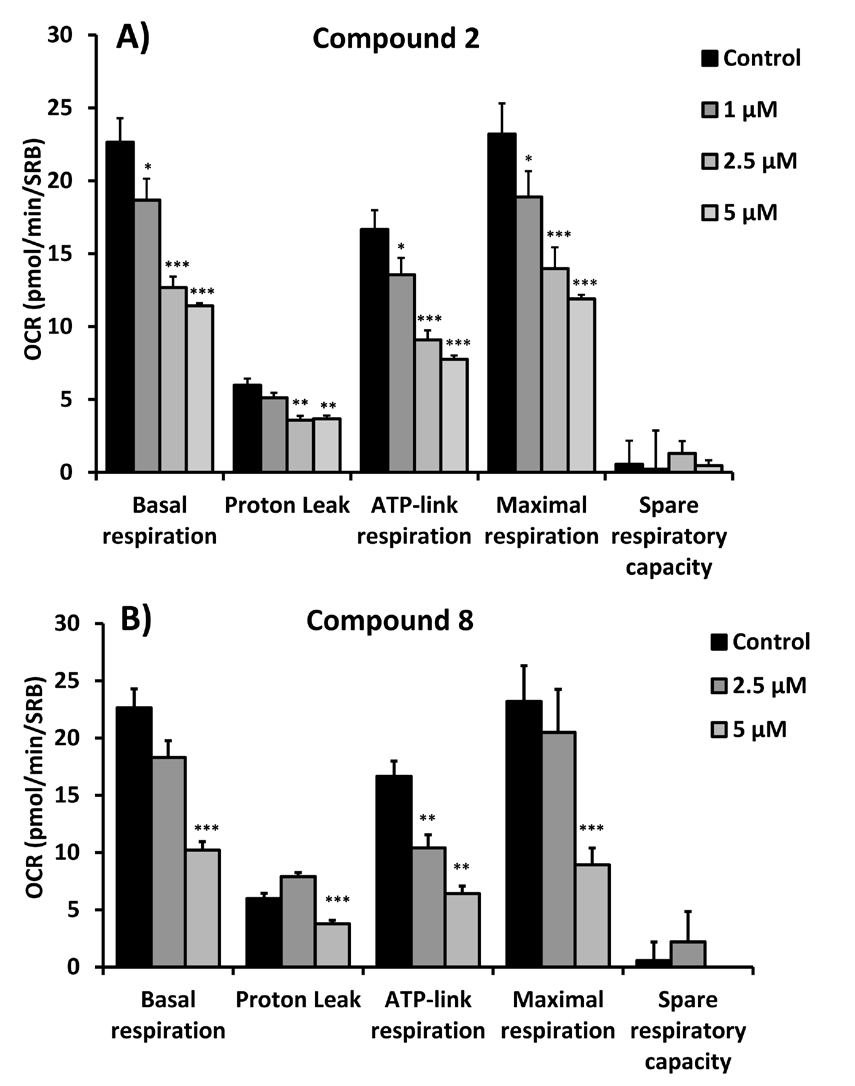
Quantitation of respiration and ATP production in MCF7 cells treated with compounds 2 (**A**) and 8 **(B)**. Their effects on basal respiration, the proton leak, ATP production, maximal respiration and spare respiratory capacity are all shown. See Figure 4 for additional details.

**Figure 6:**
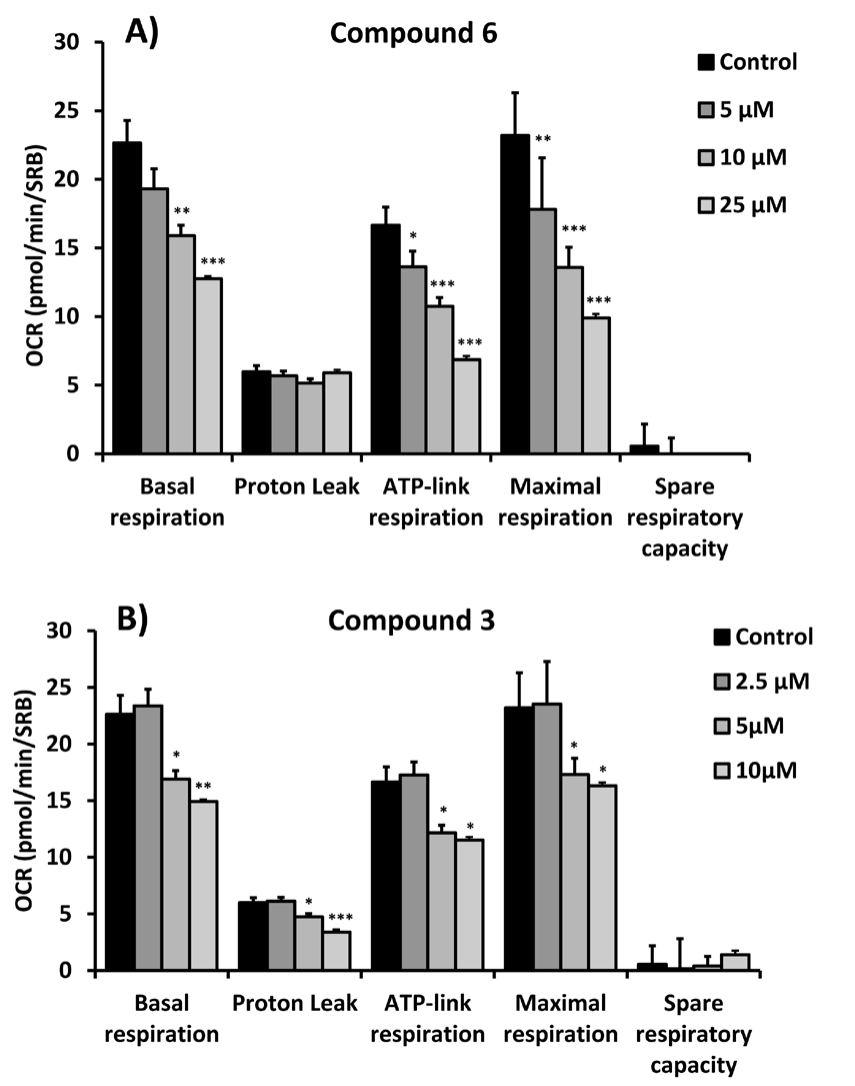
Quantitation of respiration and ATP production in MCF7 cells treated with compounds 6 (**A**) and 3 **(B)**. Their effects on basal respiration, the proton leak, ATP production, maximal respiration and spare respiratory capacity are all shown. See Figure 4 for additional details.

**Figure 7:**
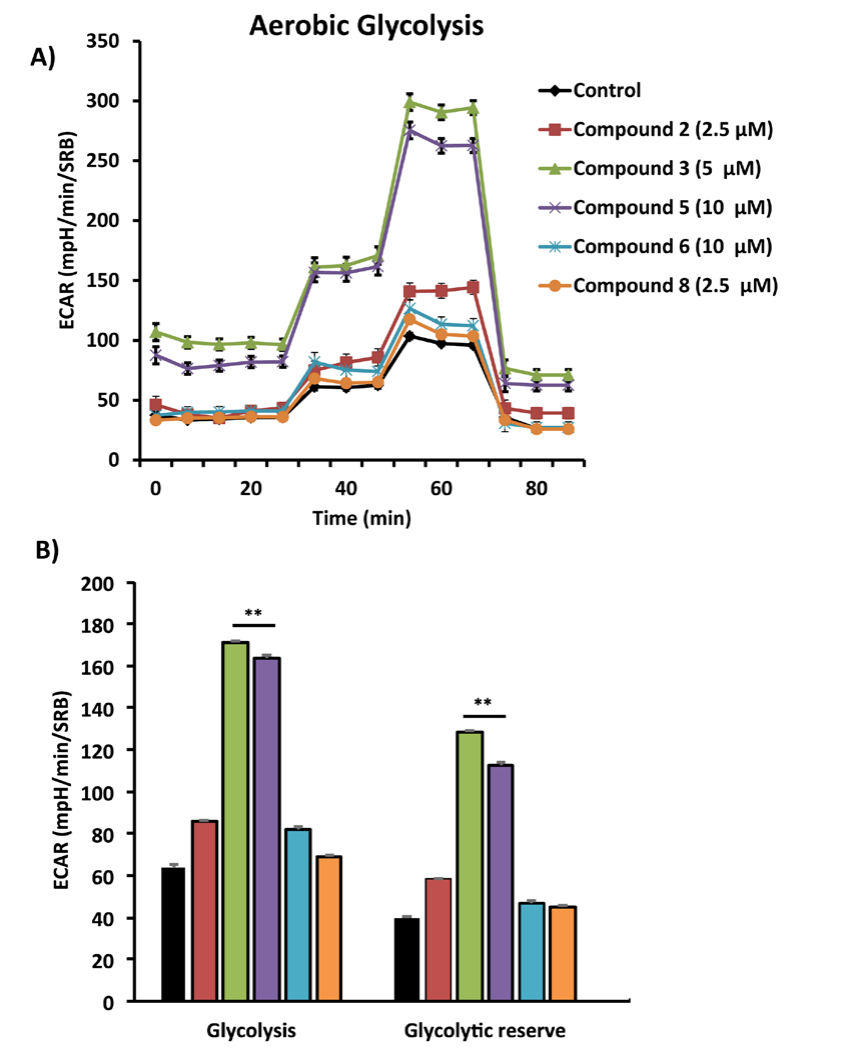
Comparative metabolic flux analysis of the top 5 hit compounds in MCF7 cells Extracellular acidification rate (ECAR) was measured using the Seahorse XFe96 Metabolic Flux Analyzer (**A**). Then data were normalized to protein content (SRB assay) (**B**). Note that compound 3 and 5 treatment stimulated glycolysis significantly, at the concentrations displayed in the graph. MCF7 cells were treated with each compound for 72 hours.

Figures 4, 5 and 6 show that compounds 2, 3, 6 and 8 all successfully targeted mitochondrial oxygen consumption (OCR} as predicted, driving ATP depletion, as measured by using the Seahorse XF metabolic flux analyzer. In addition, compounds 3 and 5 significantly stimulated aerobic glycolysis in the cancer cells (ECAR; Figure 7).

### Molecular modeling of hit compounds: docking within the active catalytic sites of OXCT1 and ACAT1

**Figure 8:**
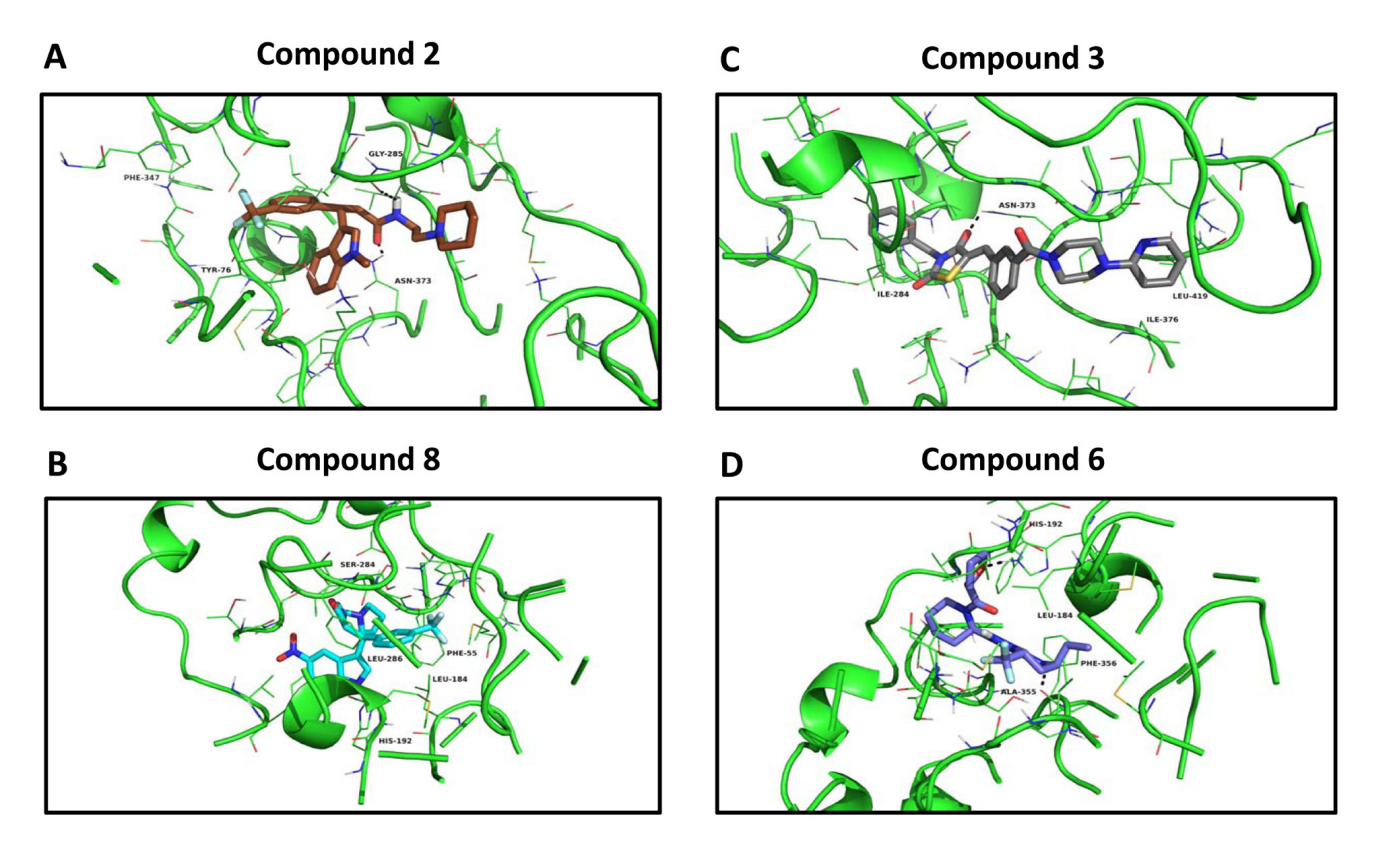
Docking images of the top 4 hits Compound 2 docking at the succinyl-CoA binding site of 3-oxoacid CoA-transferase 1 (OXCT1) (Panel **A**); compound 8 docking at the CoA binding site of human acetyl-CoA acetyltransferase (ACAT1) (Panel **B**). Compound 3 docking at the succinyl-CoA binding site of 3-oxoacid CoA-transferase 1 (OXCT1) (Panel **C**); compound 6 docking at the CoA binding site of human acetyl-CoA acetyltransferase (ACAT1) (Panel **D**).

Figure 8 illustrates the predicted binding sites of the top four hit compounds and their binding partners.

More specifically, we show the docking predicted, using the vHTS program eHiTS, of compounds 2 and 3 at the succinyl-CoA binding site within the 3D crystal structure of OXCT1. Similarly, we depict the predicted docking of compounds 6 and 8, at the CoA binding site of 3D crystal structure of ACAT1.

This type of key structural and topological information will be important for the further optimization of these hit compounds, for the more potent targeting of OXCT1 and ACAT1, in future studies.

## DISCUSSION

### Discovery of the mitoketoscins: targeting mitochondrial OXCT1 and ACAT1

**Figure 9:**
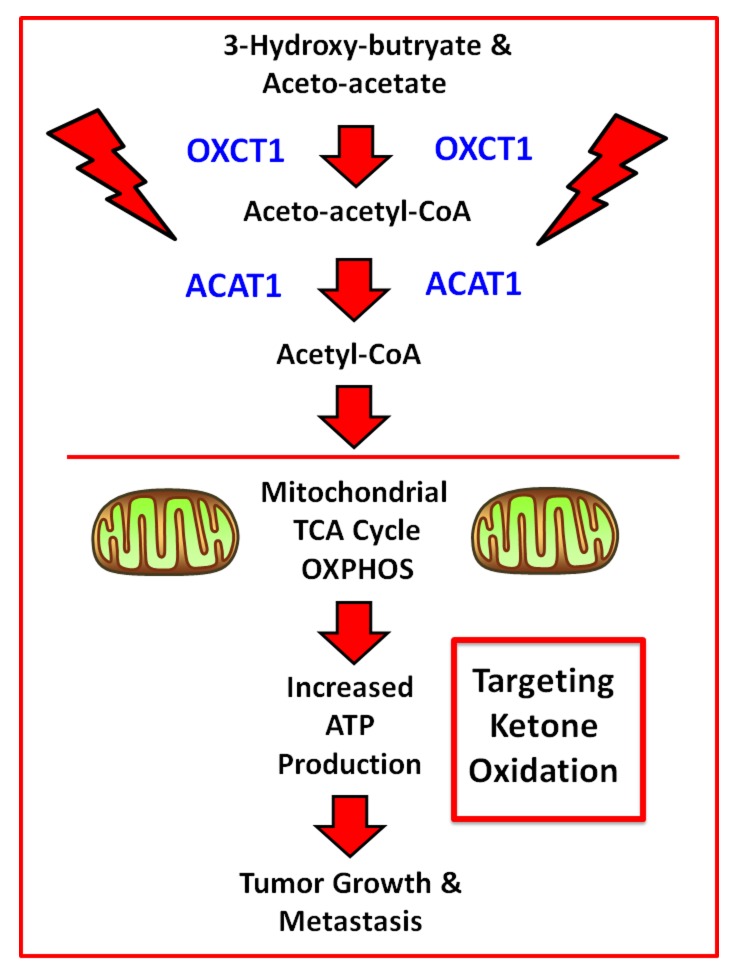
Schematic diagram summarizing how OXCT1 and ACAT1 are thought to fuel mitochondrial metabolism Note that OXCT1 and ACAT1 functionally convert ketone bodies (3-hydroxy-butyrate and aceto-acetate) into Acetyl-CoA, which can then enter the mitochondrial TCA cycle, driving ATP production. As such, ketone bodies fuel tumor growth and metastasis, via mitochondrial metabolism.

Ketone bodies functionally behave as mitochondrial fuels, which can actively drive tumor growth and metastasis. In this context, OXCT1 and ACAT1 are two mitochondrial proteins that participate in ketone re-utilization (summarized in Figure 9). For example, recombinant transduction of human breast cancer cells (MDA-MB-231) with either OXCT1 or ACAT1 is indeed sufficient to metabolically promote tumor growth and metastasis, in pre-clinical models. Based on these findings, we proposed that they function as “metabolic oncogenes”. In this report, we molecularly targeted OXCT1 and ACAT1, to prevent cancer cells from recycling ketone bodies into Acetyl-CoA, which normally enters the TCA cycle, driving mitochondrial ATP production. More specifically, we employed an *in silico* drug design approach (computational chemistry) to identify a sub-set of molecules that chemically fit within the active site of OXCT1 or ACAT1, as determined based on their published 3D crystal structures.

**Figure 10:**
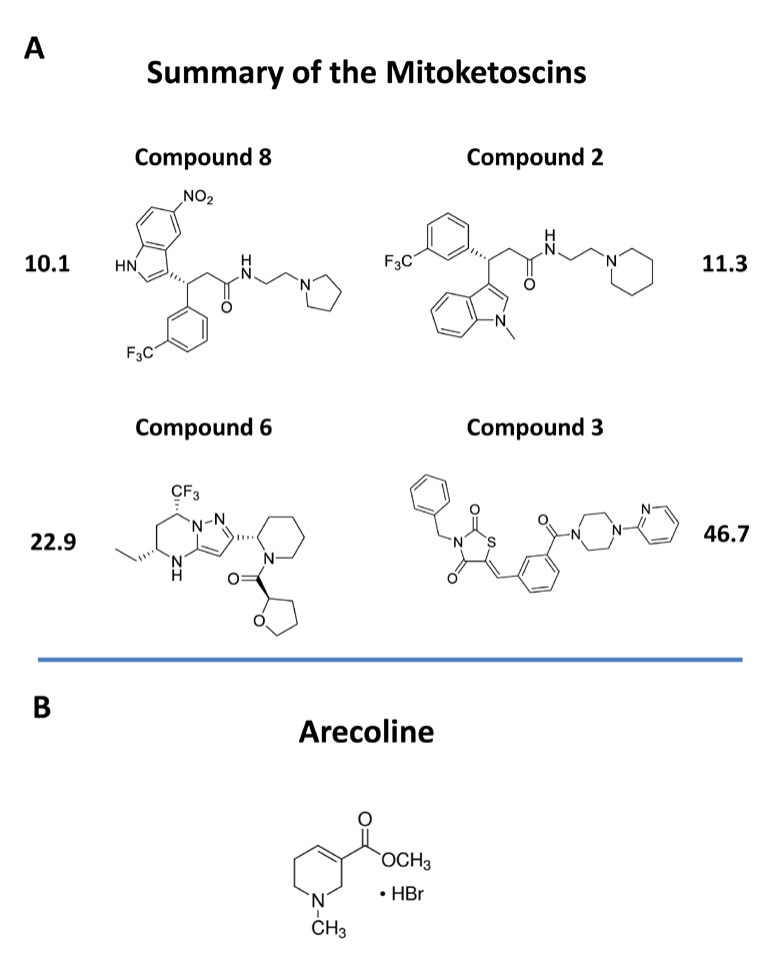
Chemical structures of novel OXCT1 and ACAT1 inhibitors **A.** The 4 top positive hit compounds and their IC-50’s for inhibiting CSC propagation are shown. **B.** The chemical structure of arecoline is shown for comparison. Arecoline is a naturally occurring ACAT1 inhibitor.

These compound libraries were then subjected to phenotypic drug screening to identify molecules that selectively induce ATP depletion. The resulting positive hits were functionally validated further, by using the Seahorse XF, to quantitatively measure mitochondrial oxygen consumption (OCR) and glycolytic (ECAR) rates. Importantly, four of the positive hits inhibited mitochondrial oxidative metabolism and two of the positive hits also induced a reactive increase in aerobic glycolysis. The overall chemical structures of these four positive hits are shown in Figure 10. Similarly, we used the mammosphere assay to assess cancer stem cell activity. Importantly, we showed that these four hit compounds significantly inhibit CSC propagation, with IC-50’s in the micro-molar range (10 and 170 μM). Our molecular modeling studies also reveal how these small molecules presumably bind, within the active site of OXCT1 and ACAT1. As such, we believe that OXCT1 and ACAT1 are critical “druggable” targets, for continued therapeutic development. Here, we propose the new term “mitoketoscins”, to reflect that these small molecules were specifically designed to target ketone re-utilization and to ultimately block mitochondrial metabolism. However, we more broadly define “mitoketoscins” as any small molecule(s) or peptide(s) that bind to ACAT/OXCT gene family members and, as a consequence, inhibit mitochondrial function, i.e., ACAT/OXCT inhibitors.

**Figure 11:**
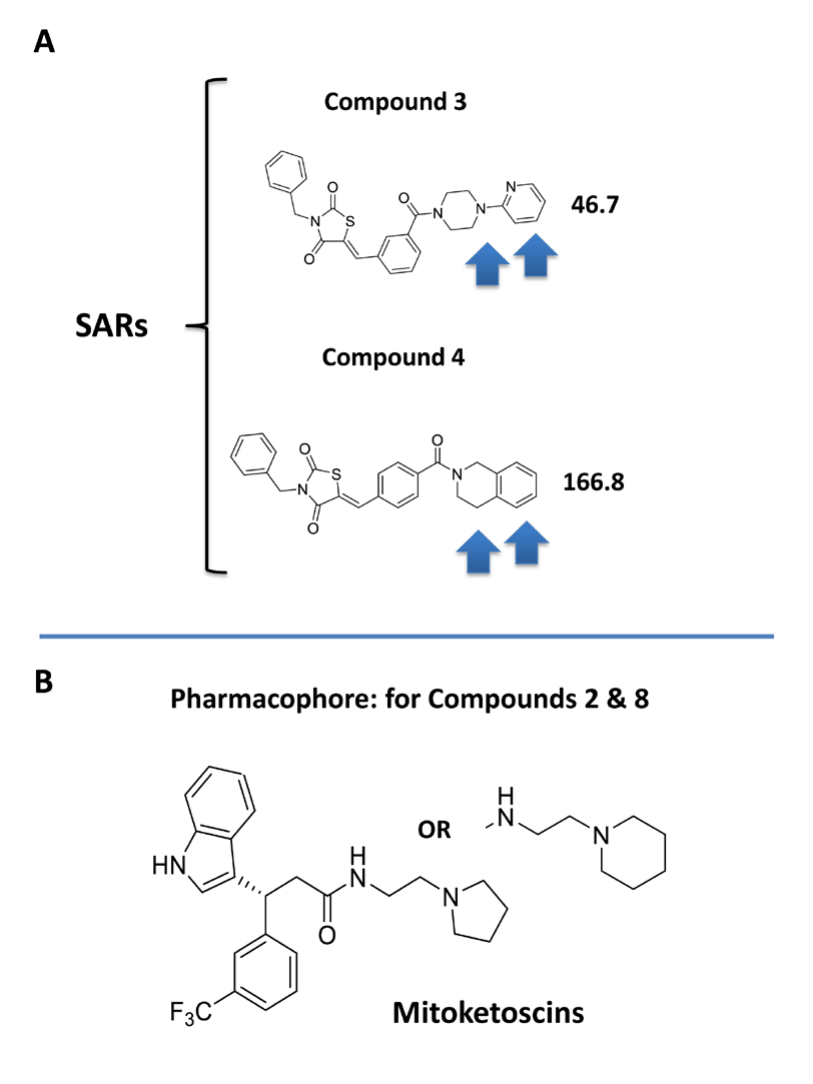
Structure activity relationships (SAR) for compounds 3 and 4 **A.** Note that compounds 3 and 4 are nearly structurally identical, but compound 3 is approximately 4 times more potent for targeting CSC propagation. Arrows highlight their structural differences. **B.** The pharmacophore for compounds 2 & 8 is shown for comparison.

**Figure 12:**
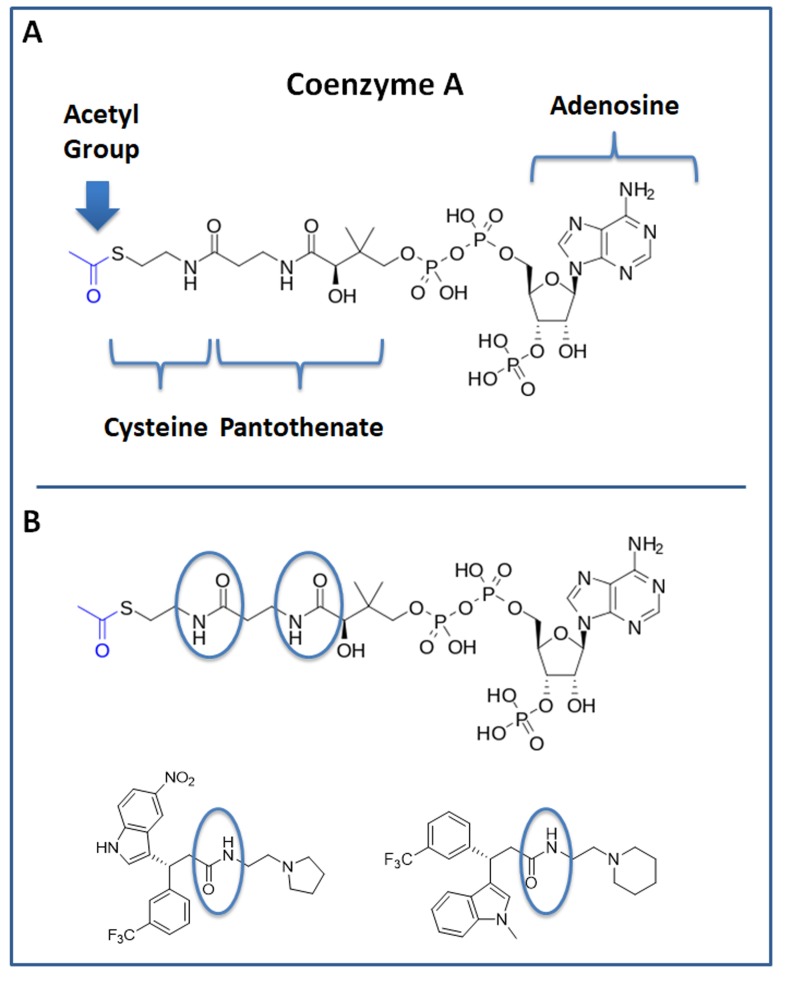
Structure of Coenzyme A (CoA) and comparison with hit compounds 2 and 8 **A.** The structure of CoA is shown. Note that it consists of cysteine, pantothenate (vitamin B5), adenosine and a series of phosphate groups. A blue arrow points at the Acetyl-group of Acetyl-CoA. **B.** The structure of CoA is shown and is compared with top hit compounds 2 and 8. An area of potentially interesting structural similarity is highlighted, using blue ovals.

Interestingly, our top hit for the OXCT1 screen (compound 2) and our top hit for the ACAT1 screen (compound 8) are nearly identical chemically, with the exception of minor functional side groups (Figure 10). So, in essence, the underlying “chemical scaffold” or *pharmacophore* is the same for both of these small molecules (Figure 11). This is perhaps not surprising since OXCT1 and ACAT1 are two enzymes within the same metabolic pathway, and they both handle very similar metabolic substrates. However, it is actually quite encouraging that using these two independent and divergent approaches (OXCT1 versus ACAT1 vHTS) that two structurally similar small molecules are functionally selected by phenotypic screening from these two different starting points. Compound 2 was initially selected for its ability to bind “in silico” to the succinyl-CoA binding site in OXCT1. Similarly, Compound 8 was originally selected for its ability to bind “in silico” to the CoA binding site in ACAT1. Therefore, the most likely possibility is that these two inhibitors are somehow chemically mimicking the structure of Coenzyme A. The structure of compounds 2 and 8 is also compared to the molecular structure of Coenzyme A (CoA; Figure 12), which illustrates some recognizable similarities.

In addition, two of the molecules from the OXCT1 screen (compounds 3 and 4) are chemically similar to each other (Figure 11). However, based on their observed IC-50 values, compound 3 is nearly 4 times more potent than compound 4, in its ability to target CSC propagation. The unique chemical groups that distinguish these two molecules structurally (highlighted by arrows), may therefore be responsible for the observed differences in their IC-50’s observed for their inhibition of CSC propagation. This structure-activity relationship (SAR) will undoubtedly facilitate and allow for further optimization of compound 3.

### Arecoline, a natural ACAT1 inhibitor

In a recent report, Fan, Chen and colleagues provided additional evidence of a role for ACAT1 as an oncogene, using pre-clinical models [9,10]. Moreover, they screened a chemical library containing mainly FDA-approved drugs and identified arecoline, a natural product as a potential ACAT1 inhibitor [9,10]. Arecoline is a nicotinic acid-based alkaloid found within the areca nut, which is the fruit of the areca palm tree (Areca catechu) [11]. In certain Asian countries, arecoline is administered by chewing the areca nut, together with the betel leaf (like chewing tobacco), as it behaves as a natural CNS stimulant [11]. Importantly, this natural product, arecoline, showed anti-tumor activity, further validating that drugs targeting ACAT1 might be valuable as anti-cancer agents [9,10]. However, the authors did not assess its capacity to target CSCs. As arecoline is very small molecule (shown in Figure 10B for comparison), it would need to be modified significantly by medicinal chemistry to increase its potency.

### Ketone bodies as a mitochondrial fuel source for CSCs

Why would CSCs favor the exploitation of ketone bodies over other energy-rich metabolic substrates for oxidation? The reason may be related to oxidative stress. Ketone bodies are metabolized by mitochondria, under conditions that dramatically reduce and/or minimize ROS production [12-17]. This may be especially beneficial to CSCs, as they are already under a significant amount of “oncogenic stress”, due to the activation of multiple oncogenic driver mutations and the loss of tumor suppressor function, resulting in increased oxidative stress. Thus, the utilization of ketone bodies may provide CSCs with a significant amount of relief/protection against ROS production, thereby preventing the onset of oncogene-induced senescence. As such, the use of ketone bodies as a fuel source may help “shield” CSCs against an overwhelming amount of mitochondrial-based oxidative stress. Thus, removal of this “shield” against endogenous ROS, by employing ACAT1 and OXCT1 inhibitors, would be expected to contribute significantly to the eradication of CSCs.

**Figure 13:**
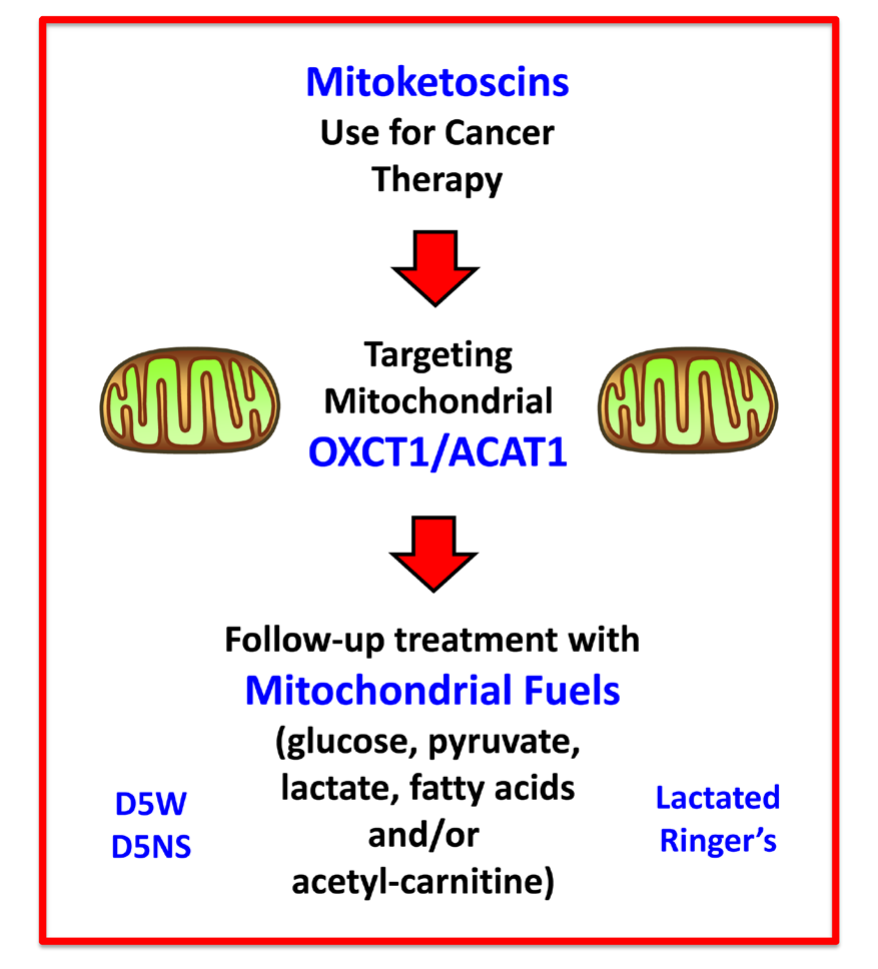
Mitoketoscin therapy: Possible follow-up treatment with mitochondrial substrates The potential side-effects of ketone inhibitors could be significantly ameliorated or “controlled” by including a “rescue” step, consisting of follow-up treatment with other mitochondrial substrates, such as glucose, pyruvate, lactate, fatty acids and/or acetyl-carnitine. Importantly, the idea of this follow-up treatment is extremely feasible, because sterile D-glucose and L-lactate i.v. solutions (D5W, D5NS, Lactated Ringer’s) are currently used routinely in hospitals for various clinical indications.

It is important to note that while normal ketone metabolism occurs strictly under conditions of organismal starvation and/or severe nutrient deprivation, this regulation is lost in human tumors, and ketone metabolism appears to occur constitutively in cancer cells. Therefore, the targeting of ketone metabolism in human tumors, under normal dietary conditions, would be predicted to have minimal metabolic side effects. Also, the potential side-effects of ketone inhibitors could be significantly ameliorated or “controlled” by including a “rescue” step, consisting of a follow-up treatment with other mitochondrial substrates, such as glucose, pyruvate, lactate, fatty acids and/or acetyl-carnitine (Figure 13). Sterile D-glucose and L-lactate i.v. solutions (D5W, D5NS, Lactated Ringer’s) are already used routinely in hospitals for other clinical and therapeutic indications, making the idea of a follow-up treatment extremely feasible.

## CONCLUSIONS

In summary, here we used a computational chemistry approach, together with phenotypic drug screening and metabolic flux analysis, to develop and functionally validate novel small molecule inhibitors of OXCT1 and ACAT1. Their ability to functionally target CSC propagation was directly validated using the mammosphere assay, as a “classic” measure of stem cell activity. Molecular modeling revealed how we believe that these small molecules fit within the active site of these enzymes, which will allow for further modification and refinement of their structures by medicinal chemistry approaches.

## MATERIALS AND METHODS

### Materials

MCF7 cells were purchased from the ATCC (American Type Culture Collection). Gibco-brand cell culture media (DMEM) was purchased from Life Technologies. The top 8 hit compounds were custom-synthesized and purchased from Avistron Chemistry Inc. (United Kingdom).

### Virtual high-throughput screening (vHTS)

Compounds were selected from a screening collection of 30,000 compounds. Initial virtual high-throughput screening (vHTS) used the eHiTS screening program [18] to identify the top 1,000 ranked compounds both for the OXCT1 and ACAT1 screen based on predicted binding affinity to the succinyl-CoA: 3-ketoacid CoA transferase from pig heart covalently bound to CoA (PDB code 3OXO) or based on predicted binding affinity to the CoA binding site of human mitochondrial acetoacetyl-CoA thiolase (PDB code 2F2S). Consensus scoring of these top 1,000 compounds was carried out using AutoDock 4.2 [19] followed by further analysis of predicted binding affinity and visual inspection using the de novo design program SPROUT [20]. Compounds performing well in all analysis steps were selected for assay. A total of 227 compounds performing well in these analysis steps were then selected for phenotypic drug screening. In summary, 84 out of 30,000 compounds (0.0028 or 0.28%) were selected for the OXCT1-based phenotypic screen. Similarly, 143 out of 30,000 compounds (0.0048 or 0.48%) were selected for the ACAT1-based phenotypic screen.

### Phenotypic drug screening, with a metabolic ATP-depletion assay

MCF7 cells (6,000 cells/well) were plated into black clear-bottom 96-well plates and incubated overnight before treatment. The resulting 227 compounds first identified by vHTS (84 compounds for the OXCT1 screen and 143 compounds for the ACAT1 screen); were then subjected to phenotypic drug screening at a concentration of 20 μM, to identify which compounds functionally induce ATP-depletion, before inducing cell death. Subsequently, positive hits were re-screened at lower concentrations (10 μM), to identify the top 8 compounds that most potently induced ATP-depletion. Compounds were tested after 72 hours of incubation and experiments were performed in duplicate. After treatment, media was aspirated from the wells and plates were washed with warm PBS (supplemented w/ Ca^2+^ and Mg^2+^). Then, cells were incubated with a Hoechst 33342 (Sigma) staining solution (10 µg/ml) for 30 min and washed with PBS (to estimate cell viability). Fluorescence was read with a plate reader using excitation/emission wave-lengths at 355/460-nm. Then, the CellTiter-Glo luminescent assay (Promega) was performed to measure metabolic activity (ATP content) in the very same wells that were treated with a given compound. Assays were performed according to the manufacturer’s protocol. Fluorescence intensity (Hoechst staining) and luminescence intensity (ATP content) was normalized to vehicle-alone treated controls and were displayed as percent control for comparison.

### Cell viability assay

The Sulphorhodamine (SRB) assay is based on the measurement of cellular protein content. After treatment for 72h in 96-well plates, cells were fixed with 10% trichloroacetic acid (TCA) for 1h in the cold room, and were dried overnight at room temperature. Then, cells were incubated with SRB for 15 min, washed twice with 1% acetic acid, and air dried for at least 1h. Finally, the protein-bound dye was dissolved in a 10 mM Tris, pH 8.8, solution and read using the plate reader at 540-nm.

### Mammosphere formation assays

A single cell suspension of MCF7 cells was prepared using enzymatic (1x Trypsin-EDTA, Sigma Aldrich) and manual disaggregation (25 gauge needle) [21]. Cells were then plated at a density of 500 cells/cm^2^ in mammosphere medium (DMEM-F12/ B27 / 20-ng/ml EGF/PenStrep) in non-adherent conditions, in culture dishes coated with (2-hydroxyethylmethacrylate) (poly-HEMA, Sigma). Cells were grown for 5 days and maintained in a humidified incubator at 37°C at an atmospheric pressure in 5% (v/v) carbon dioxide/air. After 5 days in culture, spheres >50 μm were counted using an eye-piece graticule, and the percentage of cells plated which formed spheres was calculated and is referred to as percent mammosphere formation, normalized to vehicle-alone treated controls. Mammosphere assays were performed in triplicate and repeated three times independently.

### Seahorse XFe96 metabolic flux analysis

Extracellular acidification rates (ECAR) and real-time oxygen consumption rates (OCR) for MCF7 cells were determined using the Seahorse Extracellular Flux (XF96) analyzer (Seahorse Bioscience, MA, USA) [22-25]. MCF7 cells were maintained in DMEM supplemented with 10% FBS (fetal bovine serum), 2 mM GlutaMAX, and 1% Pen- Strep. 5,000 cells per well were seeded into XF96-well cell culture plates, and incubated overnight at 37°C in a 5% CO2 humidified atmosphere. After 24h, cells were treated with the top four hit compounds at various concentrations (or vehicle alone). After 72h of treatment, cells were washed in pre-warmed XF assay media (for OCR measurement, XF assay media was supplemented with 10mM glucose, 1mM Pyruvate, 2mM L-glutamine and adjusted at pH 7.4). Cells were then maintained in 175 μL/well of XF assay media at 37°C, in a non-CO2 incubator for 1h. During incubation, 25 μL of of 80mM glucose, 9μM oligomycin, 1M 2-deoxyglucose (for ECAR measurement) and 25 μL of 10μM oligomycin, 9μM FCCP, 10μM rotenone, 10μM antimycin A (for OCR measurement) in XF assay media was loaded into the injection ports of the XFe-96 sensor cartridge. During the experiment, the instrument injected these inhibitors into the wells at a given time point, while ECAR/OCR was measured continuously. ECAR and OCR measurements were normalized by protein content (Sulphorhodamine B assay). Data sets were analyzed by XFe-96 software, using one-way ANOVA and Student’s t-test calculations. All experiments were performed in triplicate.

### Statistical analyses

Statistical significance was determined using the Student’s t-test, values of less than 0.05 were considered significant. Data are shown as the mean ± SEM, unless stated otherwise.

## Abbreviations

OXCT1: 3-Oxoacid CoA-Transferase 1 (mitochondrial)
ACAT1: Acetyl-CoA Acetyltransferase 1 (mitochondrial)
vHTS: Virtual High-Throughput Screening
